# SiN_x_/(Al,Ga)N interface barrier in N-polar III-nitride transistor structures studied by modulation spectroscopy

**DOI:** 10.1038/s41598-020-68963-7

**Published:** 2020-07-21

**Authors:** Ł. Janicki, H. Li, S. Keller, U. K. Mishra, R. Kudrawiec

**Affiliations:** 1 Department of Semiconductor Materials Engineering, Wrocław University of Science and Technology, Wybrzeże Wyspiańskiego 27, 50-370 Wrocław, Poland; 2 Department of Electrical and Computer Engineering, University of California, Santa Barbara, CA 93106 USA

**Keywords:** Semiconductors, Surfaces, interfaces and thin films, Electronic devices

## Abstract

Contactless electroreflectance studies coupled with numerical calculations are performed on in-situ SiN_x_ capped N-polar III-nitride high electron mobility transistor (HEMT) structures with a scaled channel thickness in order to analyse the built-in electric field in the GaN channel layer. The experimentally obtained field values are compared with the calculated field versus channel thickness curves. Furthermore, the experimental and theoretical sheet carrier densities, n_s_, are evaluated. While a gradual decrease in carrier concentration with decreasing channel thickness is expected for N-polar structures, experimentally a sudden drop in the ns values is observed for samples with very thin channels. The additional loss in charge was associated with a change in the SiN_x_/AlGaN interface Fermi level at very thin channel thicknesses.

## Introduction

III-N based heterostructures have been in the focus for high-frequency/power switching applications since Khan et al.^1^ demonstrated the first AlGaN/GaN high electron mobility transistor (HEMT). Because of difficulties in growth of high quality N-polar layers and heterostructures Ga-polar III-nitrides were dominant in recent years. However, N-polar HEMT structures possess certain advantages over Ga-polar ones due to the different structure geometry resulting from the reverse polarization direction that kept the pursuit of high quality growth along the (000-1) axis alive^2^. Progress in metal–organic chemical vapour deposition (MOCVD) growth allowed the achievement of smooth N-polar surfaces^3–5^. HEMTs with record power densities of 8 W/mm at 94 GHz were reported recently^6^ together with impurity concentrations as low as several 10^16^ cm^−2^ in GaN^7^.

Effective surface passivation is necessary to eliminate surface traps that otherwise hamper device parameters like two dimensional electron gas (2DEG) concentration and mobility, and for that purpose silicon nitride, SiN_x_, is often employed^8–11^. Thereby SiN_x_ grows in an amorphous phase resulting in broken interface bonds that can create interface states^12,13^. For metal polar structures a reduction of the (Al,Ga)N surface barrier height after SiN_x_ capping has been reported on both thin layer heterostructures and bulk-like layers^14–18^ and was attributed to a lowered amount of surface states (through passivation or charge neutralization) or creation of interface states which pin the Fermi level.

Because N-polar (Al,Ga)N is known to be easily oxidized^19,20^, with the effect stronger than on the Ga-polar surface, the need for surface protection is even more crucial in order to ensure a stable operation irrespectively of the environment conditions. Examining N-polar GaN/AlGaN structures with an *in-situ* MOCVD Si_3_N_4_ a barrier height of ~ 1 eV at the Si_3_N_4_/GaN interface was determined via capacitance–voltage (C–V) measurements^21^. More recently, the Fermi level at ex-situ Si_3_N_4_/AlGaN interfaces was reported to be located close to the conduction band (CB) over a broad range of Al contents in both metal- and N-polar AlGaN up to 60%^22^.

The position of the Fermi level at the surface directly affects the field in the channel of N-polar HEMT structures. As the thickness of the GaN channel is decreased for the fabrication of highly scaled devices, the field in the GaN channel increases, resulting in a decrease in channel charge when all other structure parameters are held constant^23,24^. In contrast to Ga-polar HEMT structures, in the N-polar design the decrease in charge with decreasing channel thickness is coupled with a significant reduction also in electron mobility due to enhanced scattering from charged interface states with increasing field in the channel^25^, which can lead to a significant increase in sheet resistance if not accounted for^23,24^.

Modulation spectroscopy is a technique that allows to gain insight into built-in electric fields in semiconductor structures through Franz-Keldysh oscillation (FKO) appearing in optical spectra in presence of medium to high fields^26,27^. In all of the structures in this study a built-in electric field arising from polarization properties of III-nitrides is expected to be present in subsequent layers. Of particular interest is the GaN channel built-in electric field since it influences the two dimensional electron gas (2DEG) density. This field itself is dependent not only on polarization effects but also on the surface or, in case of SiN_x_ capped structures, interface Fermi level position^28^. Therefore it seems practical to first study the Fermi level position of bare and capped structures to understand the effect of SiN_x_ capping.

In this work contactless electroreflectance (CER) spectroscopy is applied to study the built-in electric field in the GaN channel of N-polar HEMTs with different GaN channel thicknesses and cap layers. Extracted field values are then compared with numerical calculations of the GaN channel field dependency on channel thickness with varied interface Fermi level position (i.e. interface barrier height). From the comparison it is observed that a shift from 0.5 to 1.3 eV in the Fermi level position occurs when the sheet carrier density decreases beyond a critical value when reducing the channel thickness. At the same channel thickness a sudden drop in carrier concentration was observed in electrical measurements.

## Structures studied

**Figure 1:**
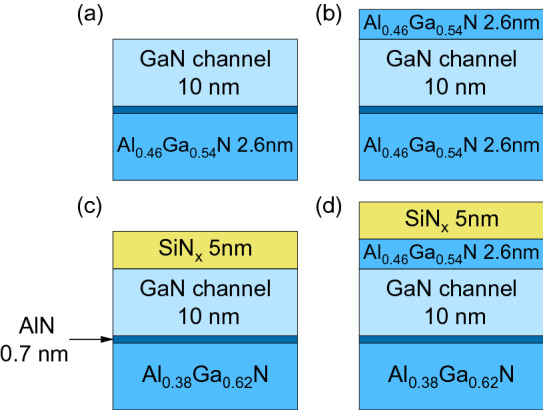
Schematics of samples from the 1st group. The samples share a common backbarrier design up to the AlN interlayer and a common GaN channel thickness. Sample (**a**) was uncapped; sample (**b**) was capped with an Al_0.46_Ga_0.54_N layer; sample (**c**) was capped with a SiN_x_ layer; sample (**d**) had both the Al_0.46_Ga_0.54_N and SiN_x_ capping layers applied. Samples from the 2nd and the 3rd series share the design with the sample (**d**), the difference being the GaN channel layer thickness and the backbarrier doping level.

Three series of structures were prepared for this study that shared a common stack design as follows: a 1.4 μm thick semi-insulating GaN base layer was deposited on a C-plane sapphire. Next a backbarrier was prepared that consisted of a 20 nm thick graded Al_x_Ga_1-x_N layer with x = 0.05 → 0.38 and a 10 nm thick Al_0.38_Ga_0.62_N film. On top of the backbarrier a 0.7 nm thick AlN interlayer was introduced to decrease carrier scattering. Finally a GaN channel layer of varied thickness was deposited and capped with a 2.6 nm thick Al_0.46_Ga_0.54_N cap layer (absent in two of the 1st series structures). As a dielectric a SiN_x_ film was introduced on top. Details on individual samples are given in Table 1. Samples were grouped in three series. The 1st series with and without the top AlGaN and/or SiN_x_ layers was prepared to serve as a reference series that provided a comparison between capped and uncapped structures, sample stacks are shown in Fig. 1a–d. The 2nd and 3rd series were grown for carrier concentration studies and they share the design with the stack shown in Fig. 1d but with a varied GaN channel layer thickness and additional backbarrier doping.

**Table 1:** GaN channel thicknesses, AlGaN top layer thicknesses, SiN_x_ cap layer thickness, silicon doping level in backbarrier, and measured 2DEG carrier concentration for the investigated samples from the three series.

	Sample	d_GaN_^a^ (nm)	d_AlGaN_^b^ (nm)	d_SiNx_^c^ (nm)	n_Si_^d^ (×10^18^ cm^−3^)	n_S_^e^ (×10^12^ cm^−2^)
1st series	1	10	0	0	4	n/a
	2	10	0	5	4	n/a
	3	10	2.6	0	4	n/a
	4	10	2.6	5	4	n/a
2nd series	1	20	2.6	5	4	9.84
	2	15	2.6	5	4	8.42
	3	10	2.6	5	4	Resistive
3rd series	1	12	2.6	5	5	9.99
	2	10.5	2.6	5	5	9.36
	3	9	2.6	5	5	3.19

The backbarrier design was the same for all samples.

^a^GaN channel thickness.

^b^AlGaN cap thickness.

^c^SiN_x_ thickness.

^d^Silicon doping in backbarrier.

^e^Measured 2DEG carrier concentration in the GaN channel.

## Results and discussion

Hall measurement results obtained for samples from 2nd and 3rd series, presented in Table 1, show a predictable decrease in carrier concentration with narrowing of the GaN channel when the thickness is changed from 20 to 15 nm (2nd series) or from 12 to 10.5 nm (3rd series). However, in both series, the concentration suddenly drops 3 times (3rd series) or becomes unmeasurable (2nd series). The sudden increase in resistivity was previously associated with the circumstance, that in N-polar GaN/AlGaN heterostructures the decrease in charge with decreasing channel thickness is coupled with a significant reduction in electron mobility.

**Figure 2:**
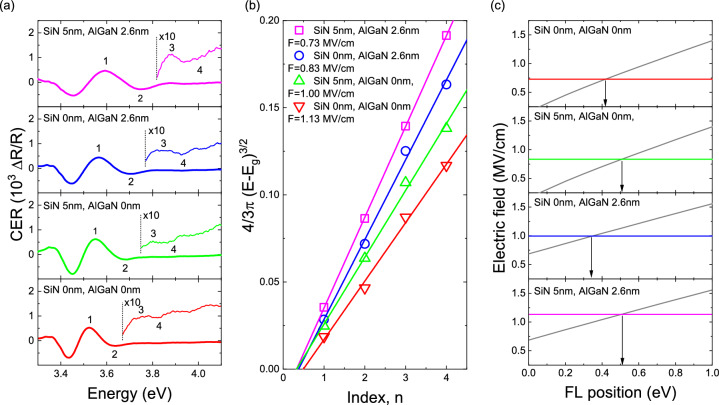
CER spectra with subsequent FKO numbered recorded for the 1st series of samples (**a**) and an analysis of the built-in electric field in each GaN channel of each structure (**b**). In panel (**c**) the calculated curves of the field dependency on the surface (interface—in case of SiN_x_ capped structures) Fermi level are shown and compared with experimentally obtained field values (horizontal lines) to extract the actual surface/interface barrier, shown with arrows.

Since the field in the GaN channel strongly depends on the Fermi level position on the outer boundary of a structure, be it its surface or an interface with a capping dielectric, first samples with and without SiN_x_ and/or Al_0.46_Ga_0.54_N layers were studied by CER. Figure 2a shows spectra recoded for the 1st series of structures. A band-to-band transition followed by FKO originating from the GaN channel is visible in CER spectra recorded for all samples. Clearly, FKO extrema shift towards higher energies with addition of Al_0.46_Ga_0.54_N and/or SiN_x_ indicating an increase in field values. The assessment of the field, shown in Fig. 2b, was done in a conventional way by analysing the energetic position of FKO extrema^26,27^. Without SiN_x_ and Al_0.46_Ga_0.54_N layers the built-in electric field in the GaN channel layer is 0.73 MV/cm. An addition of SiN_x_ and/or Al_0.46_Ga_0.54_N causes the field to change due to a shift of the surface/interface Fermi level and/or creation of polarization-induced charges. In the SiN_x_ capped structure a slight increase in the field to 0.83 MV/cm is observed. Fields values in structures with the Al_0.46_Ga_0.54_N layer, both capped and uncapped, is also higher at 1.13 MV/cm and 1.00 MV/cm, respectively.

In order to translate the obtained field values to barrier height numerical calculations of dependency of built-in electric field on the surface/interface Fermi level position were performed, the results are shown in Fig. 2c. Crossings of calculated curves with horizontal lines indicating experimental field values show the respective Fermi level position. It can be seen that for GaN a surface barrier of ~ 0.4 eV is observed. This is a similar value to 0.3 eV reported previously for air ambient exposed N-polar GaN^28–30^. Quite unexpectedly a lower surface barrier of ~ 0.35 eV is estimated for the uncapped Al_0.46_Ga_0.54_N terminated structure. In ultra-high vacuum (UHV) conditions a higher initial barrier for GaN and a gradual increase in surface barrier in AlGaN alloys were observed by x-ray photoelectron spectroscopy (XPS) previously^22^. The discrepancy may be related to surface oxidation of samples under study within this work and N-polar nitrides are known to be easily oxidated^30^. SiN_x_ capping shifts the Fermi level slightly away from the CB edge in both structures with and without the Al_0.46_Ga_0.54_N layer to ~ 0.5 eV. Such an effect of SiN/AlGaN interface barrier stabilisation was reported in Ref.^22^ and, since for SiN_x_ capped structures ambient composition should not have any effect, a comparison between aim ambient (here) and UHV conditions (Ref.^22^) is not unjustified.

**Figure 3:**
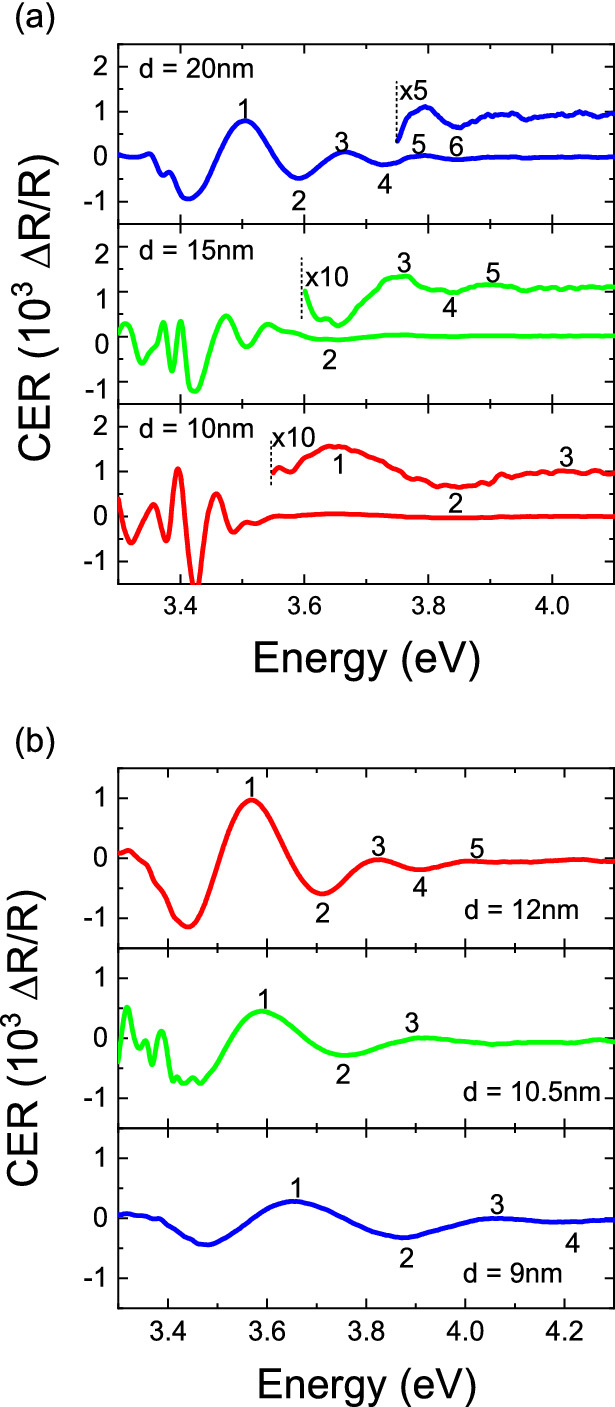
CER spectra with subsequent FKO numbered recorded for the 2nd (**a**) and 3rd (**b**) series of samples.

**Figure 4:**
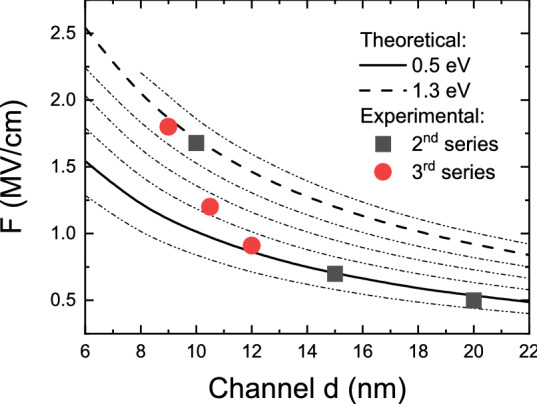
Calculated dependency of the built-in electric field on GaN channel thickness for various surface barrier heights. Superimposed are experimentally obtained field values for samples from the 2nd (squares) and 3rd (circles) series. A clear shift of the surface barrier at ~ 10 nm is visible.

Similar CER studies were performed for both 2nd and 3rd series, consisting of full structures with a top Al_0.46_Ga_0.54_N layer and capped by SiN_x_ that were previously studied by Hall effect measurements. The resulting CER spectra with FKO extrema marked are shown in Fig. 3a,b, respectively. In both series an expected increase in FKO period, i.e. increase of the built-in electric field, can be seen with narrowing of the GaN channel. Analysis of FKO yielded field values of 0.50, 0.71, and 1.68 MV/cm for structures with 20, 15, and 10 nm GaN channel thickness (2nd series) and 0.91, 1.20, and 1.80 MV/cm for structures with 12, 10.5, and 9 nm GaN channel thickness (3rd series). It can be immediately noticed that the field increase between 15 and 10 nm (10.5 and 9 nm) is much steeper than between 20 and 15 nm (12 and 10.5 nm) channel thickness. To better understand the observed effect a dependency of channel field on channel thickness was calculated for SiN_x_/Al_0.46_Ga_0.54_N barrier of 0.5 eV and several other values. It can be seen in Fig. 4 that the experimental points follow the 0.5 eV line only up to a certain thickness of 10 nm where a jump occurs to ~ 1.3 eV for two structures that showed lowered or unmeasurable carrier concentration. However, the channel thickness itself cannot be a factor that causes such a drastic change in surface barrier height and, in turn, carrier concentration. N-polar HEMTs are known to be highly scalable with carrier concentrations in excess of 10^13^ cm^−2^ even with channel thickness below 6 nm^23,31^ and, therefore, a different mechanism must be responsible for the observed carrier concentration drop.

**Figure 5:**
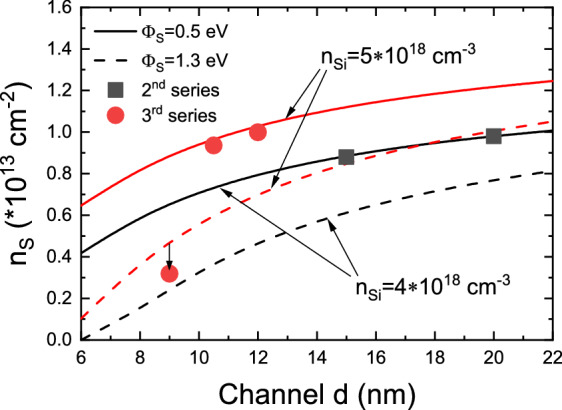
Calculated dependency of the carrier concentration *n*_*s*_ on GaN channel thickness for two doping concentrations of 4 × 10^18^ cm^−3^ and 5 × 10^18^ cm^−3^ representing actual doping levels in 2nd and 3rd series samples. Superimposed are experimentally obtained carrier concentrations for samples from the 2nd (squares) and 3rd (circles) series. Below ~ 8 × 10^12^ cm^−2^ carrier concentration a sudden decrease is observed resulting from the SiN_x_/AlGaN interface barrier increase.

Having established the SiN_x_/Al_0.46_Ga_0.54_N interface Fermi level position for all samples, calculations of the carrier concentration dependency on the GaN channel thickness were performed and are shown in Fig. 5. Two interface barrier heights were selected that correspond to the ones deducted above, namely 0.5 eV and 1.3 eV, and for each barrier two curves were calculated with doping level in the barrier of 4 × 10^18^ cm^−3^ and 5 × 10^18^ cm^−3^ that correspond to doping levels in 2nd and 3rd series, respectively. Results of Hall measurements of four samples that show high carrier concentration nicely follow the calculated curves for a barrier height of 0.5 eV following a gradual decreasing carrier concentration with narrowing of the GaN channel. The predicted carrier concentration for the 9 nm sample (3rd series) at a barrier height of 0.5 eV is 0.87 × 10^13^ cm^−2^. The experimentally obtained carrier concentration of 3.19 × 10^12^ cm^−2^ is, however, even lower than the calculated value of 4.66 × 10^13^ cm^−2^_._ Regarding the 10 nm sample (2nd series) that was too resistive for Hall measurements its predicted carrier concentration at barrier of 0.5 eV was 0.71 × 10^13^ cm^−2^, and at 1.3 eV, a barrier that corresponds to CER measurements, calculations show 0.33 × 10^13^ cm^−2^. As was observed for the former sample the real concentration is probably even lower causing the Hall measurement to be unsuccessful.

In order to understand this change in barrier height a discussion of previous reports is necessary. Two interface barrier heights were reported for N-polar SiN_x_/GaN. In Ref.^22^ it was estimated by XPS that the barrier is ~ 0.3 eV in case of bulk-like films . An earlier paper on SiN_x_ passivated GaN/AlGaN heterostructures reports a ~ 1.0 eV barrier deducted from C-V studies^21^. At the same time the latter report gives an insight on the SiN_x_/GaN interface state density providing a value of 4.5 × 10^12^ cm^−2^ and stating that this interface charge is contained within 0.21 eV around the 1.0 eV surface state. These results suggest that two separate surface states exist at the SiN_x_/Al_0.46_Ga_0.54_N interface. Here we propose a model that describes the rapid decrease in carrier concentration for channel thicknesses below a “critical” value based on filling of these levels by carriers and subsequent changes in the band diagrams.

**Figure 6:**
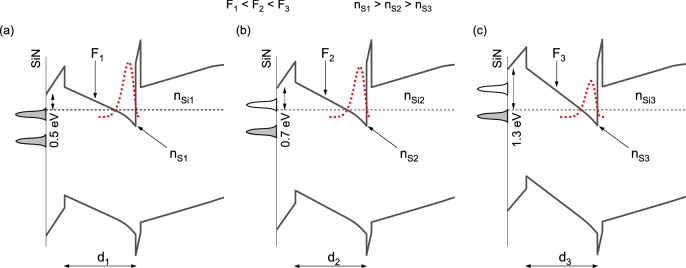
Band profiles near the SiN_x_/AlGaN interface calculated for three cases: (**a**) Fermi level set firmly in the upper interface state, (**b**) intermediate case with the Fermi level shifted towards the bottom of the upper surface state, and (**c**) Fermi level shifted to the lower surface state. For each case a surface barrier is given. Indicated are built-in electric field *F*, GaN channel thickness *d*, backbarrier doping level *n*_*Si*_, and a resulting 2DEG concentration *n*_*S*_.

Figure 6 shows three cases of surface state occupancy and resulting band profiles of a N-polar HEMT structure identical to the ones studied experimentally within this paper with a GaN channel thickness and built-in electric field *d* and *F*, respectively, and a barrier doping level *n*_*Si*_. Two surface states are considered. In the first case the interface barrier is set at 0.5 eV, i.e., in the upper interface state. In the intermediate case a slight increase in the barrier height is depicted that results from a downward Fermi level shift within the upper state. The last case shows a band diagram that results from a further downward shift of the interface Fermi level to the lower interface state at 1.3 eV. At constant *d* and *n*_*Si*_ the field values are *F*_*1*_ < *F*_*2*_ < *F*_*3*_. Carrier concentration follows with *n*_*S1*_ > *n*_*S2*_ > *n*_*S3*_. Now a situation of decreasing *d* at constant *n*_*Si*_ will be considered. With a narrowing of the channel the field *F* increases and the separation between carriers filling the upper surface state and the triangular potential well (TPW) at the GaN/AlGaN interface decreases promoting a carrier transfer from the surface state towards TPW. At a certain point the upper interface state is depleted of carriers hence the Fermi level shifts to the lower state. This in turn causes a significant change in the GaN channel band bending (i.e., the field increases) and a subsequent drop in *n*_*s*_. While it may seem that in the intermediate case *n*_*s*_ should increase due to carrier transfer it actually decreases because of gradually increasing GaN channel field. The second case to consider is decreasing *n*_*Si*_ at a constant thickness *d*. With a decrease in doping concentration there will be less carriers in the channel while the TPW itself does not change. Empty states in TPW will attract carriers from the upper interface state causing them to migrate along the built-in electric field. Again there will be initial gradual change in the surface barrier height within the upper interface state range of energies and a subsequent shift to the lower state when all carriers are removed from the upper one.

Comparing the model and experimental data for both the 2nd and 3rd series reducing the channel thickness results in a gradual reduction in carrier concentration down to a certain thickness. Below that thickness a sudden drop of the measured *n*_*s*_ values below the predicted ~ 0.7 × 10^12^ cm^−2^ or ~ 0.9 × 10^12^ cm^−2^, respectively, assuming a Fermi level position of 0.5 eV, can be observed. At the same time, a change in the interface barrier was observed for the two samples with the narrowest channel in respective series. Taking *n*_*Si*_ as a variable one may compare the 10.5 nm sample from the 3rd series and the 10 nm sample from the 2nd series. While the channel thickness is not identical it is very close and the only significant difference is the doping level in the barrier at 5 × 10^18^ cm^−3^ for the 3rd series sample and 4 × 10^18^ cm^−3^ for the 2nd series sample. It can be seen from Hall measurements that a higher backbarrier doping allows the 3rd series to maintain a high carrier concentration while the other one shows a complete collapse. The proposed model allows also to explain why the 2nd series 15 nm structure maintains a high carrier concentration at 0.87 × 10^13^ cm^−2^ while the 3rd series 9 nm structure does not keep its predicted concentration of similar 0.88 × 10^13^ cm^−2^. The reason here is the difference in the GaN channel built-in electric field. For a narrower channel and the interface barrier of 0.5 eV the calculated field is 1.1 MV/cm, compare to actual 0.7 MV/cm seen experimentally in the structure with a 15 nm channel. A higher field will provide more potential for the interface carriers to migrate towards TPW.

Basing on the proposed model the apparent discrepancy in the SiN_x_/GaN interface Fermi level position reported in Refs.^21^^,^^22^ can be explained. In a bulk-like material studied in Ref.^22^ only a weak surface band bending exists that results from the interface states occupancy by carriers coming from the bulk. In this case the upper state is at least partially filled by electrons originating from the unintentional background *n*-type doping that is common in N-polar GaN. This results in a low surface barrier of ~ 0.3 eV. The much higher interface barrier of 1.0 eV reported in Ref.^21^ results from the built-in electric field present in GaN/AlGaN/GaN structures that draws the SiN_x_/GaN electrons towards the potential well present at the GaN/AlGaN interface emptying the upper interface state and shifting the Fermi level to the lower one.

In order to better understand the mechanism of Fermi level switching between two SiN_x_/(Al)GaN interface states more studies are needed to determine e.g. the density of interface states and their origin. It also seems important to fully describe the conditions at which the surface barrier stays low ensuring a high 2DEG concentration. Similar studies for other combinations of dielectric/III-nitride structures also seem important since various materials are proposed for gate dielectrics.

## Summary

N-polar GaN/AlGaN HEMT structures were studied by combining CER spectroscopy, Hall effect measurements, and numerical solution of Schrödinger and Poisson equations. Modulation spectroscopy provided built-in electric field values necessary to calculate accurate band profiles of heterostructures that were key in understanding of the observed phenomena. Calculated carrier concentrations compared with Hall effect experimental values provided additional confirmation of simulated band profiles. The observed sharp decrease of carrier concentration for structures with thin GaN channels was ascribed to a downward shift of the interface Fermi level between two SiN_x_/AlGaN interface states, from 0.5 to 1.3 eV below the conduction band of the top Al_0.46_Ga_0.54_N layer. Two main mechanisms are proposed for such behaviour: (1) narrowing of the channel layer that brings the triangular potential well at the channel/barrier interface closer to surface states and simultaneously increases the channel built-in electric field, and (2) a decrease in backbarrier doping that reduces the 2DEG density while leaving the potential well intact that attracts carriers from surface states.

## Methods

### Sample growth

All samples investigated in this study were grown by metal–organic chemical vapour deposition (MOCVD). C-plane sapphire substrates with 4° misorientation towards sapphire-a-plane were used to achieve smooth N-polar (Al,Ga)N films^4^. A 1.4 μm thick semi-insulating (S.I.) GaN base layer was first deposited using the procedure reported previously. For all samples, the backbarrier layer consisted of a 20 nm thick graded Al_x_Ga_1−x_N layer with x = 0.05 → 0.38 and a 10 nm thick Al_0.38_Ga_0.62_N film, followed by a 0.7 nm thick AlN interlayer, a GaN channel layer with thickness varying from 9 to 20 nm, a 0 or 2.6 nm thick Al_0.46_Ga_0.54_N cap layer, and a 0 or 5 nm thick in-situ SiN_x_ film. The graded Al_x_Ga_1−x_N layers were doped with Si to achieve n-type doping of 4 × 10^18^ cm^−2^ (1st and 2nd series) or 5 × 10^18^ cm^−2^ (3rd series). The SiN_x_ film was grown at 1,030 °C using disilane and ammonia flows of 4.46 μmol/min and 268 mmol/min, respectively.

### 2DEG concentration measurements

Van der Pauw Hall measurements with indium contacts were performed at room temperature to determine the carrier concentrations.

### Contactless electroreflectance measurements

For CER measurements the samples were mounted in a capacitor with a half-transparent top electrode made from a copper-wire mesh. An air gap of ~ 0.5 mm was kept between the sample surface and the top electrode. An alternating voltage of ~ 3 kV provided the band bending modulation. Other relevant details on CER can be found in Refs.^32,33^.

### Calculations

A commercial package nextnano++ was used for band profile and carrier concentration calculations^34^ that provides solutions for coupled Schrödinger–Poisson equations.

## Data Availability

The datasets generated during and/or analysed during this study are available from the corresponding author on reasonable request.
